# Doxorubicin-provoked increase of mitotic activity and concomitant drain of G0-pool in therapy-resistant BE(2)-C neuroblastoma

**DOI:** 10.1371/journal.pone.0190970

**Published:** 2018-01-17

**Authors:** Isabell Hultman, Linnea Haeggblom, Ingvild Rognmo, Josefin Jansson Edqvist, Evelina Blomberg, Rouknuddin Ali, Lottie Phillips, Bengt Sandstedt, Per Kogner, Shahrzad Shirazi Fard, Lars Ährlund-Richter

**Affiliations:** 1 Department of Women's and Children's Health, Karolinska Institutet, Stockholm. Sweden; 2 Department of Oncology and Pathology, Karolinska Institutet, Stockholm. Sweden; University of Navarra, SPAIN

## Abstract

In this study chemotherapy response in neuroblastoma (NB) was assessed for the first time in a transplantation model comprising non-malignant human embryonic microenvironment of pluripotent stem cell teratoma (PSCT) derived from diploid bona fide hESC. Two NB cell lines with known high-risk phenotypes; the multi-resistant BE(2)-C and the drug sensitive IMR-32, were transplanted to the PSCT model and the tumour growth was exposed to single or repeated treatments with doxorubicin, and thereafter evaluated for cell death, apoptosis, and proliferation. Dose dependent cytotoxic effects were observed, this way corroborating the experimental platform for this type of analysis. Notably, analysis of doxorubicin-resilient BE(2)-C growth in the PSCT model revealed an unexpected 1,5-fold increase in Ki67-index (p<0.05), indicating that non-cycling (G0) cells entered the cell cycle following the doxorubicin exposure. Support for this notion was obtained also *in vitro*. A pharmacologically relevant dose (1μM) resulted in a marked accumulation of Ki67 positive BE(2)-C cells (p<0.0001), as well as a >3-fold increase in active cell cycle (i.e. cells positive staining for PH3 together with incorporation of EdU) (p<0.01). Considering the clinical challenge for treating high-risk NB, the discovery of a therapy-provoked growth-stimulating effect in the multi-resistant and p53-mutated BE(2)-C cell line, but not in the drug-sensitive p53wt IMR-32 cell line, warrants further studies concerning generality and clinical significance of this new observation.

## Introduction

Childhood cancers show fundamental differences to most common adult solid tumours in their cancer-causing genetics, cell biology, and importantly also their local tissue microenvironment [1]. Neuroblastoma (NB) is the most common extracranial solid tumour during infancy, half of which are clinically manifested before the age of 18 months [2]. Moreover, evidence for early stage microscopic tumour-like NB lacking metastasis in young infants supports the notion of an origin already during the prenatal phase [3]. The diagnosis comprises a spectrum of embryonic tumours of the peripheral sympathetic nervous system and shows a high degree of intra- and inter-tumoural heterogeneity on both genetic and phenotypic levels, as well as unique abilities to spontaneously regress/differentiate or develop metastatic phenotypes [2]. Metastasis and relapse are the main causes of death, which make it imperative to understand metastatic dissemination and intra-tumour variations for therapy responsiveness. An influence from the microenvironment on clonal dominance likely contributes to the disparity between primary and metastatic tumours seen in many patients, as well as inter-tumour heterogeneity between patients with the same tumour type [4, 5]. Modelling of tumour micro colonisation reflects in this context the ability of cell subpopulations to comply or adapt to new environments (i.e. subsets of cells either present in a minority at diagnosis or develop during therapy), a feature with great impact on metastasis and clinical prognosis [6].

Several *in vivo* NB-models are available, including among others subcutaneous or orthotopic xenografts, as well as genetically modified models to simulate tumour induction and growth, ideally confined to the relevant tissue environment [7]. Patient-derived xenograft (PDX) models generated by injection of fresh human tumours to mice is widely considered a clinically more predictive alternative compared to serially transplanted tumour lines [8]. Specifically for NB, orthotopic PDX has been suggested as the model of choice for studying invasion and metastasis ([9] and ref therein).

Progress will come from deciphering the complex cross talk between the primary tumour, its immediate microenvironment, and metastatic niches. A comprehensive program to systematically evaluate anti-tumour agents for childhood cancers in various models for significant clinical activity (The Pediatric Preclinical Testing Program; supported by The National Cancer Institute) noted that the dominant difference between the gene expression of xenograft models and their human counterparts was the signature contributed by stromal cells [10]. Considering these findings it is noteworthy that a large study on colorectal cancer demonstrated that when gene expression patterns in human tissue environment from patient material were compared to results in a PDX model the analysis in mouse stroma showed significantly altered predictions on clinical response to therapy [11]. Further, convincing data today link processes of cancer progression to induction of cellular potency [12, 13]. At the same time there is an increasing insight regarding differences between human and mouse species for the signalling pathways controlling the induction of cellular potency [14–16].

Developmental and species aspects are thus of importance when analysing the relevant signalling between NB and the host. A driving momentum behind the here presented approach is that compared to current animal xeno-models, a homologous embryonic setting may provide a favourable micro-environmental setting for studies and preclinical evaluation of embryonic tumours and their response to chemotherapy. Tzukerman, Skorecki and co-workers were first to demonstrate the use of non-malignant human experimental teratoma as a more optimal niche for intercellular interaction and a tool in cancer research investigating the stromal response in human tumour cell growth [17–21]. The model represents increasingly chaotic embryonic processes, comprising compartmentalised tissues or organoid-like development including stages immediately preceding the positioning of adrenal sympatical progenitors in embryonic mesenchyme [22]. This led us to test the PSCT milieu for *in vivo* support of tumours of embryonic origin, establishing the NB-PSCT model ([23, 24] and reviewed in [25]). The embryonic nature of the model makes the approach especially applicable for so-called ‘embryonic childhood cancers’ originating early in life.

Here we apply the human embryonic PSCT experimental platform to explore chemotherapy-responsiveness of two well-characterised NB tumours.

## Material and methods

### Ethical permissions

This study was performed in strict accordance with permissions from the Local Ethics Committee at Karolinska Institute (114/00) and from the regional ethics committee (Stockholm Northern Animal Review Board; Dnr N101/13; N118/14). All surgery was performed under approved protocols for anaesthesia and all efforts were made to minimize suffering.

### Cell lines

BE(2)-C and IMR-32 were obtained from ATCC (Manassas, VA, USA). Cell line authentications were performed using STR analysis.

BE(2)-C is a clonal subline of the multidrug-resistant and p53-mutated NB tumour line SK-N-BE(2) which was derived 1972 from a metastatic site of the bone marrow from a 22-month old boy after repeated courses of chemotherapy and radiotherapy. IMR-32 was derived from a metastatic site in abdominal mass of a 13 months old boy. Both cell lines have been demonstrated to exhibit a poorly differentiated phenotype *in vivo* and genetic features typical for high-risk NB [23]. NB-cells were cultured in Thermo Scientific HyClone RPMI 1640 medium supplemented with 10% fetal bovine serum, 1% L-glutamine (Invitrogen, Carlsbad, CA, USA), and 1% penicillin/streptomycin (Invitrogen, Carlsbad, CA, USA), at 37°C, 5% CO2 with high humidity.

### Chemotherapy

Doxorubicin (doxo) (Sandoz; Ebewe) was diluted in 1xPBS (DPBS GIBCO ™ Life technology) to indicated doses and administered by intra peritoneal injections (PSCT model), or added to indicated concentrations in cell culture medium (*in vitro* analysis). Control (mock) treatment was performed with diluent (1xPBS). The *in vitro* IC50-value of doxo for IMR-32 (0.02 μM) is 400 times lower than for BE(2)-C (8.0 μM), classifying them as doxo-sensitive and doxo-resistant, respectively.

### PSCT in vivo model

Pluripotent stem cell induced experimental teratoma (PSCT) was generated in NOD SCID gamma (NSG) mice from diploid bona fide hESC as described [23, 24, 26]. In brief; 8–12 weeks old NSG male mice received an injection of 10^5^ HS181 cells (46XX) under the testicular capsule (one side) [27]. When the growth reached a diameter over 9mm, 2x10^6^ NB cells in logarithmic growth were injected in a 50μl medium suspension. Animal hosts were randomised into treatment groups (5–6 animals per group) and doxo therapy started 14 days after transplantation of NB cells.

PSCT including NB growth was harvested at indicated time points, formalin-fixed, paraffin embedded and processed as previously described [23, 24]. The blocks were consecutively cut at 4μm or 10μm and stained with Hematoxylin&Eosin (HE) for histological orientation and indicated analysis.

Positive engraftment of IMR-32 was verified by fluorescent in situ hybridization (FISH) using probes specific for human chromosome X (spectrum orange) and Y (spectrum green) (Vysis CEP X/Y DNA 30–16: nr 7J2050 Abbott). The presence of human Y-chromosome was taken as evidence for tumour cells, growing in female (46XX) teratoma environment (PSCT). The simultaneous signal for the X-chromosome was used as internal control of the assay. Positive engraftment of BE(2)-C, lacking a stable Y-chromosome centromere, was verified by FISH using probe for detection of amplified NMYC (spectrum orange) (Vysis LSI N-MYC so 3219; nr 5J5001 Abbott).

The histological slides were scanned in a Hamamatsu 2.0 high-resolution scanner and analysed using the NDP.view2 software. The percentage of positive cells was determined through blind assessment by two well-trained researchers. The immunohistochemistry (IHC) results were verified using positive control slides from tissues known to express the antigen of interest. Negative controls included using tissues known to be negative for the marker, omitting primary antibody and the use of isotype control antibody. A list of used antibodies is presented in Table 1.

**Table 1:** Antibodies and kits used for immunohistochemistry.

Antigen	Source	Dilution	2:nd detection
Ki67	Abcam, AB:833.500. Rabbit pk ab. Lot 713408	1:50	Vectastain Universal Elite DAB-ABC, or goat anti rabbit Cy3.5 1:300
Cleaved caspase 3 (c-casp3)	BD-Pharamingen, Purified rabbit anti-active caspase-3, Cat: 559565	1:1000	Vectastain Universal Elite ABC; or goat anti rabbit Cy3.5 1:300
Histone H3 phosphorylated at serine 3 (PH3)	Merck, Purified rabbit Anti-phospho-Histone H3 Cat: 06–570	1:2000	Goat anti rabbit AF488 1:1000
5-ethynyl-2′-deoxyuridine (EDU)	Click-iT™ EdU Alexa Fluor™ 488 Imaging Kit, C10337; Invitrogen™	According to manufacturer´s protocol	NA
TUNEL	DeadEnd Flourometric TUNEL, (G3250, Promega)	According to manufacturer´s protocol	NA

Proliferation was assessed using IHC for the expression of Ki67 [28]. Early apoptosis was assayed using IHC staining for cleaved caspase 3 (c-casp3). Cell death was assayed by morphological appearance of mitotic catastrophe, i.e. multiple micronuclei and nuclear fragmentation visualized by DAPI staining [29].

### In vitro analysis

NB cells in logarithmic growth were suspended and dispersed into petri dishes containing glass-coverslips (VWR #631–0149). For BE(2)-C: 2x10^5^ cells, and for IMR-32: 1x10^6^ cells, were added per plate (Day 0). A minimum of three separate experiments were performed, each experiment in duplicates.

Doxo was added to a concentration of 1μM (Day 1). For mock-treatment, the same amount of 1xPBS was added. Forty-eight hours later (Day 3), the cells were fixed for 15 min in ice-cold freshly prepared 4% paraformaldehyde (then washed and stored in 1xPBS at +4°C); or alternatively received a second treatment (1μM doxo or 1xPBS) given 1 hour after replacement of medium (allowing culture condition to recover before adding the chemotherapy). The cells were then cultured for another 48 hours before fixation (Day 5). Forty-eight hours before fixation 1μM of EdU was added to each plate.

Immunocytochemistry was performed accordingly; following fixation, cells were incubated in TNB Buffer: 0.5g of blocking reagent (#FP1020) to 100 ml TBS buffer (Tris/NaCl pH 7.4) for 30 min at room temperature. Cells were then incubated with the 1´ab diluted in 0.3% TX-100, 0.1% NaN_3_ in 1xPBS over night at +4°C. Following washing, cells were incubated with 2´ab diluted in TNB buffer, for 2 h at room temperature. The cells were washed and mounted with Prolong Gold anti-fade with DAPI to visualize nuclei. Stained cells were analysed using a Metafer® Slide Scanning Platform.

A list of the antibodies and kits used is presented in Table 1.

### Statistical analysis

Collected data was analysed using one-way analysis of variance (Anova). Bonferroni correction or Tukey´s multiple comparison post-hoc test was applied to adjust for multiple testing. The software GraphPad Prism 6.0 or 7.03 was used for testing normal distribution and generation of graphs. The *in vitro* data was analysed in RStudio and percentage of positive cells followed by the mean (±SEM) for each combination of labelling was calculated. All scores presented in percentages were transformed into rationalized-arcsine-units [30], the transformation of data makes the distribution normal, thereby reducing problems related to the use of a percentage-scale.

## Results

### A. In vivo analysis

#### Micro colonisation of BE(2)-C and IMR-32 in the PSCT model

**Fig 1:**
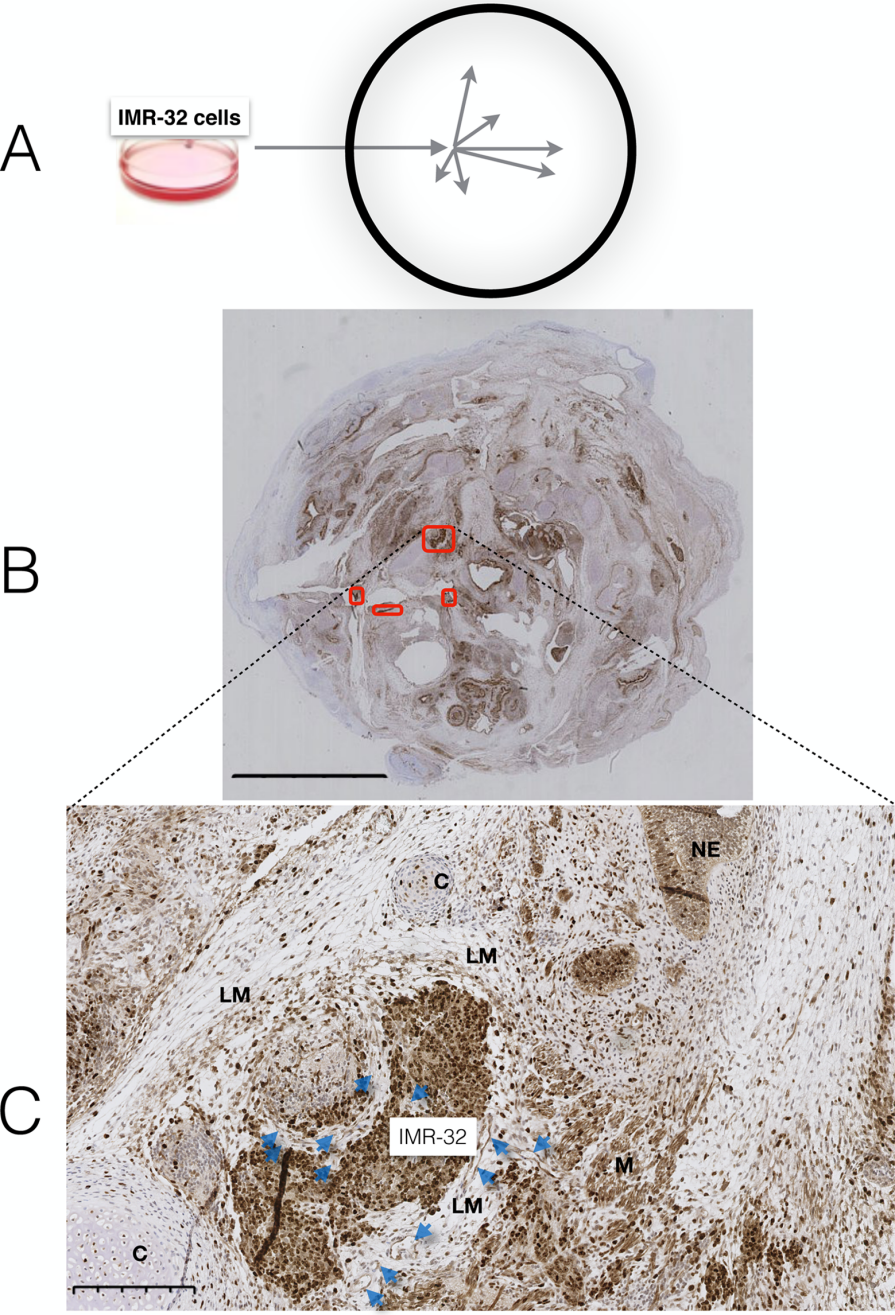
Micro colonisation of IMR-32 tumours in the PSCT model. (A) Schematic illustration; NB cells were injected into an arbitrary position centrally in the PSCT cellular mass, resulting in multiple micro-colonisations from migrating NB cells. (B) A representative FFPE section of a PSCT with four IMR-32 colonies indicated (red borders). (C) IMR-32 colony surrounded by loose mesenchyme. LM = loose mesenchyme; NE = neural epithelium; C = cartilage; M = muscle; Blue arrows = vessels. Size bars: B:5mm, C:500μm.

Tumour growth could be observed two weeks after transplantation of BE(2)-C or IMR-32 cells to the PSCT model, in line with previous reports (24). Micro-colonies were initiated by migrating NB-cells showing tropism to loose mesenchyme in immediate proximity of blood vessels, the preferred cellular context/niche supporting micro-colonisation (Fig 1) (24).

#### Doxo-induced cell death of BE(2)-C and IMR-32 in the PSCT model

**Fig 2:**
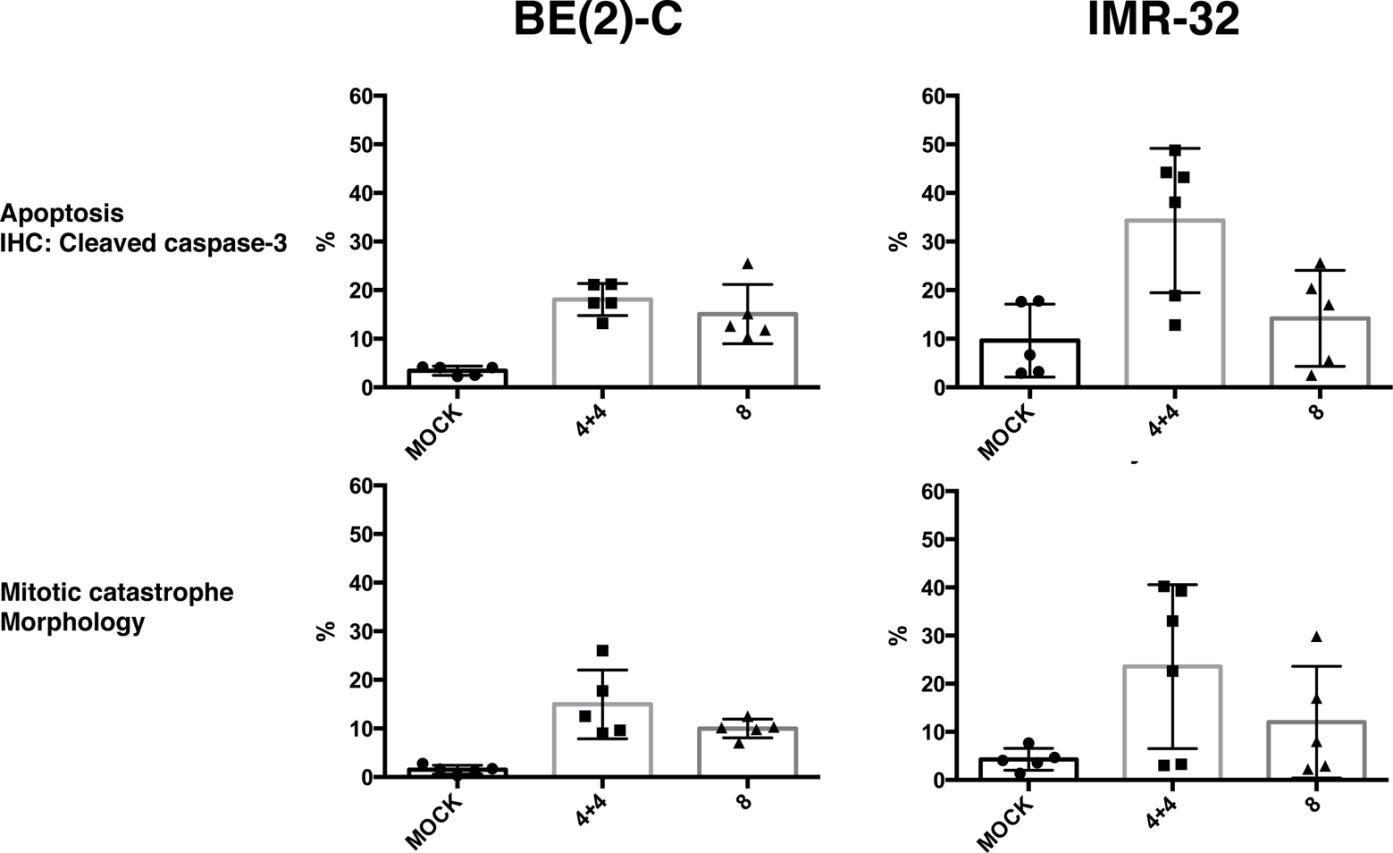
The effects of doxo on BE(2)-C and IMR-32 tumours in the PSCT model. Fraction of cells (%; mean±SD) expressing indicated markers following indicated treatments. mock = PBS; 4+4 = Repeated dose 4+4mg/kg doxo with 48h interval; 8 = single dose 8mg/kg doxo. Effects measured 96h after first administration of doxo. Data based on 5 PSCT per group. For statistical analysis, see text.

Next, doxo was administered to the mouse host by single or repeated intraperitoneal injections as described below and illustrated in Tables 2 and 3 and Fig 2. Considering the reported multi-resistance and high doxo-IC50 value of BE(2)-C [31], we titrated intraperitoneal regimens with host sub-lethal doses of doxo, aiming to attain significant levels of cytotoxicity (Table 2). A dose of 4mg/kg did not result in significant levels of cell death in BE(2)-C cells. Raising the dose to 8mg/kg resulted in a significantly higher mitotic catastrophe compared to mock-treatment. Repeated doses of 4 + 4mg/kg given with a 48 hour interval increased the frequency of mitotic catastrophe in BE(2)-C cells (Table 2, Fig 2). Besides mitotic catastrophe we were interested in investigating early apoptosis. Positive staining for c-casp3 was used as an indication of early apoptosis. The 8mg/kg dose resulted in a significantly higher value compared to mock-treatment in BE(2)-2 cells. Similar levels of c-casp3 were observed after repeated doses (4 + 4mg/kg) as with a single dose (8mg/kg) (Table 2). Based on these findings, the 8mg/kg and 4 + 4mg/kg regimens were chosen for further studies.

**Table 2:** The effects of doxo on BE(2)-C tumours growing in the PSCT model.

	BE(2)-C						
	mock	doxo		doxo		doxo	
Marker		4mg/kg	*p*	4+4mg/kg	*p*	8mg/kg	*p*
Mitotic catastrophe	1,5 ± 0,9	2,9 ± 0,9	NS	15 ± 7,1	***	10 ± 1,9	**
c-casp3	3,5 ± 1,0	NT		18 ± 3,3	***	15 ± 6,1	**
Ki67	43 ± 6,1	NT		64 ± 14	**	49 ± 6,2	NS

Fraction of cells (%; mean±SD) expressing indicated markers 96 hours following indicated treatments. Data based on 5 PSCTs per group. Mock = diluent (1xPBS). NT = not tested. Statistical comparisons between the mock- or doxo-treated group: NS = p>0.05

** = p<0.01

*** = p<0.001.

**Table 3:** The effects of doxo on IMR-32 tumours growing in the PSCT model.

	IMR32				
	mock	doxo		doxo	
Marker		4+4mg/kg	*p*	8mg/kg	*p*
Mitotic catastrophe	4,3 ± 0,2	28 ± 15	**	12 ± 12	NS
c-casp3	9,6 ± 7,5	39 ± 12	**	14 ± 9,9	NS
Ki67	63 ± 8,3	67 ± 9,0	NS	59 ± 17	NS

Fraction of cells (%; mean±SD) expressing indicated markers 96 hours following indicated treatments. Data based on 5 PSCT per group. Mock = diluent (1xPBS). Statistical comparisons between the mock- or doxo-treated group: NS = p>0.05

** = p<0.01.

Administration of 8mg/kg doxo resulted in 12±12% cells presenting mitotic catastrophe and 14±9.9% cells staining positive for c-casp3 in IMR-32 cells. This was not significantly different from mock-treatment (Table 3, Fig 2). Repeating the dose (4 + 4mg/kg doxo given with a 48 hour interval) resulted in 28±15% presenting mitotic catastrophe (not significantly different from mock-treatment: 4.3±0.2%) and 39±12% cells staining positive for c-casp3 (mock-treatment: 9.6±7.5%; p<0.05) (Table 3) in IMR-32 cells. Notably, a high variability in therapy response between individual PSCTs was observed for these tumours (Fig 2), affecting the statistical outcome.

#### Effects on proliferation of BE(2)-C and IMR-32 in the PSCT model

**Fig 3:**
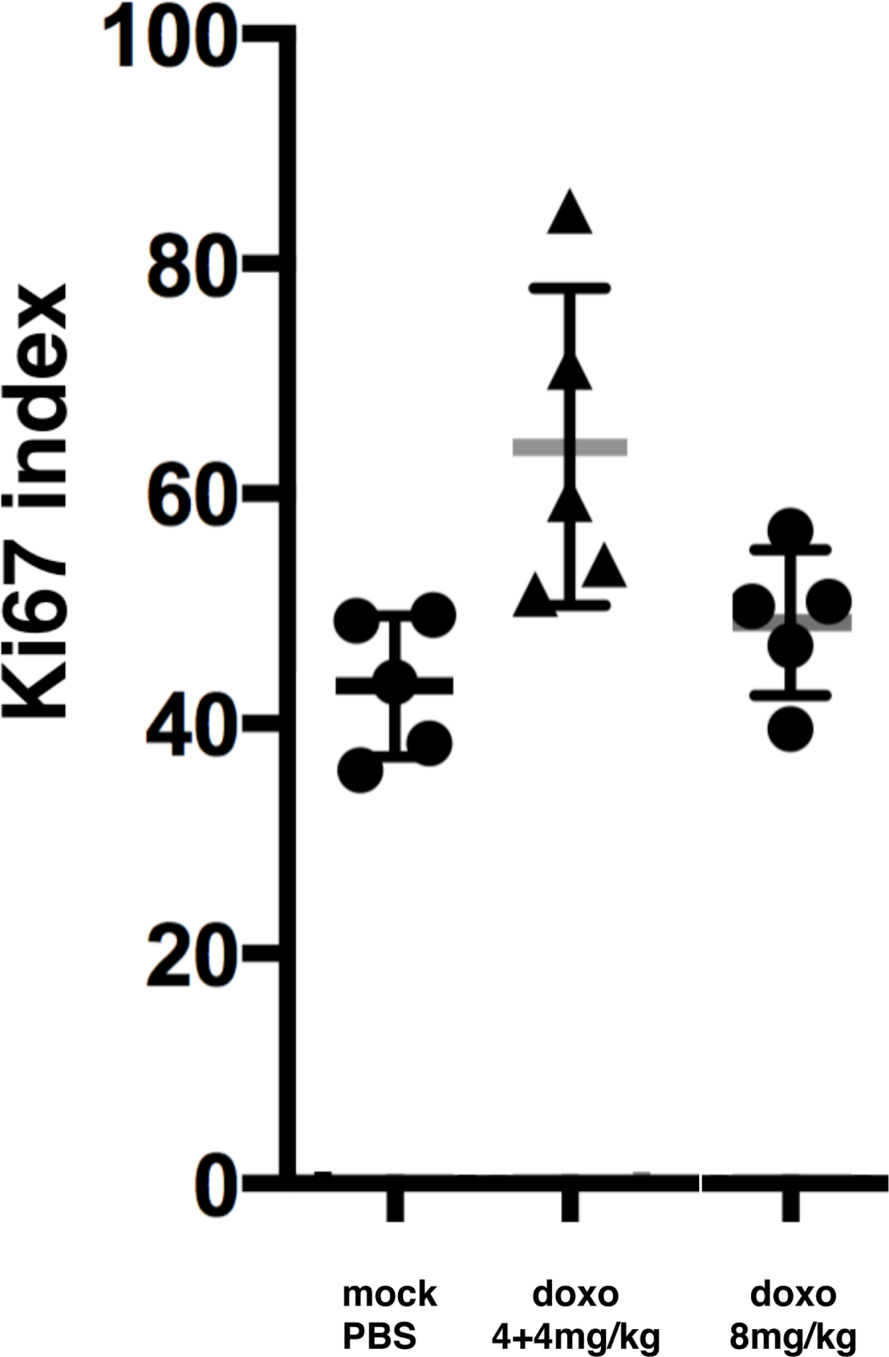
Percentage Ki67 positive cells in BE(2)-C tumour following doxo treatment in the PSCT model. Fraction of cells (%; mean±SD) expressing Ki67 96 hours after indicated treatments. Mock = diluent (1xPBS). Data based on 5 PSCT per group. For statistical analysis, see text.

BE(2)-C tumours in PSCT exhibited a Ki67-index of 43 ± 6.1%, similar to our earlier findings [20]. A single administration of 8mg/kg doxo did not result in altered Ki67-index (Table 3 and Fig 2), However, repeated treatment (4+4mg/kg) resulted in a significant increase of Ki67-index; from 43% to 64% (p<0.01): (Table 3 and Fig 3).

IMR-32 tumours in PSCT exhibited a Ki67-index of 63±8.3%, similar to previous findings [20], and this proliferative index was not significantly altered by either of the administered doses of doxo, when compared to control (Table 3, Fig 3).

#### Effects on the PSCT microenvironment

PSCT-tissues with high mitotic activity were tested for doxo-induced toxicity, as an indication of toxic side effects in adjacent non-malignant tissues. The Ki67-index in proliferative neural epithelium was not significantly affected following treatment with 8mg/kg or 4+4mg/kg doxo (Table 4). Low levels of early apoptosis (c-casp3) were indicated in NE following either treatment regimes (3.3±1.7% and 3.0±3.0%, respectively; Table 4). Frequencies of mitotic catastrophe however showed no significant differences in treated compared to mock-treated tumours (Table 4).

**Table 4:** The effects of doxo in neural epithelium in the PSCT model.

	Neural epithelium				
	mock	doxo		doxo	
Marker		4+4mg/kg	*p*	8mg/kg	*p*
Mitotic catastrophe	0.6 ± 0.7	0.4 ± 0.3	NS	0.1 ± 0.3	NS
c-casp3	0.7 ± 1.1	3.3 ± 1.7	**	3.0 ± 3.0	*
Ki67	36 ± 8.0	40 ± 15	NS	37 ± 12	NS

Fraction of cells (%; mean±SE) expressing indicated markers 96 hours following indicated treatments. Mock = diluent (1xPBS). Data based on 5 PSCTs per group. Statistical comparisons between the mock- or doxo-treated group: NS = p>0.05

* = p<0.05

**; = p<0.01.

**Fig 4:**
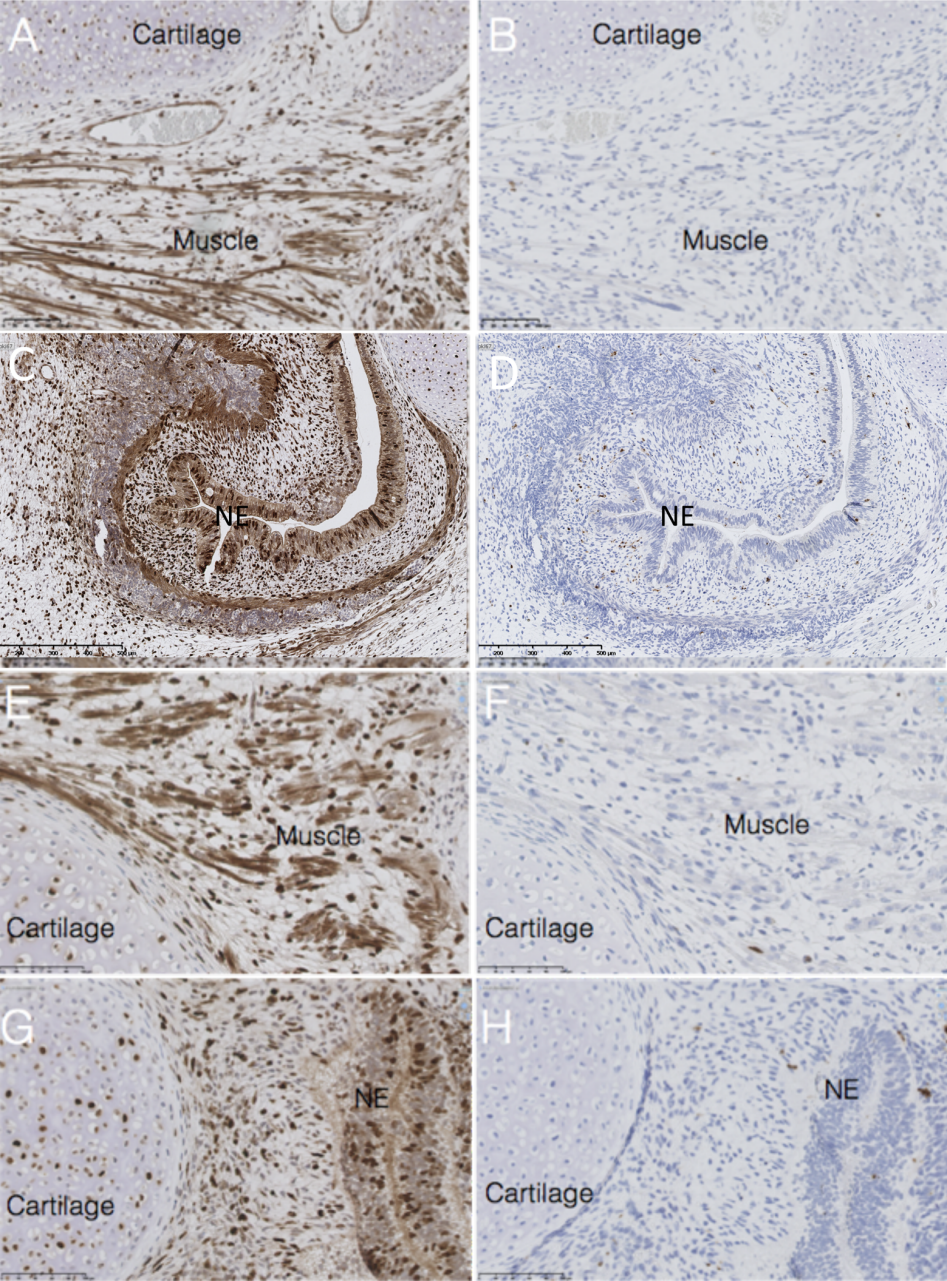
The effects of doxo on PSCT non-malignant embryonic tissues. Immunohistochemistry staining of formalin-fixed paraffin-embedded PSCT histological slides, following intra peritoneal injection of the host mouse with 8mg/kg doxo (A-D), or 4+4mg/kg doxo (E-H). High frequencies of positive staining for Ki67 in tissues compatible with neural epithelium, muscle and cartilage can be seen, indicative of extensive proliferation (A,C,E,G). Low frequencies of positive staining for cleaved caspase 3 can be seen in NE, muscle and cartilage, indicative of low frequencies of apoptosis (B,D,F,H). Size bars: 50μm.

Embryonic cartilage and embryonic muscle are additional examples of components exhibiting strong proliferation in PSCT [26, 32]. Fig 4 illustrates a typical staining for c-casp3 in embryonic cartilage and embryonic muscle, 96 hours after administration of doxo, illustrating low or absent apoptosis.

### B. In vitro analysis

Tables 5 and 6 summarise the *in vitro* data for BE(2)-C and IMR-32, respectively.

**Table 5:** Data set for BE(2)-C *in vitro*.

	BE(2)-C 48h				BE(2)-C 96h			
	mock		doxo		mock		doxo	
Marker			1 μM	*p*			1+1 μM	*p*
TUNEL	0.1 ± 0.1	≃	0.2 ± 0.1	NS	0.0 ± 0.0	≃	0.6 ± 0.8	NS
c-casp3	3.7 ± 1.7	≃	3.6 ± 0.5	NS	5.3 ± 0.1	≃	8.0 ± 2.6	NS
Ki67	36 ± 5.5	<	97 ± 1.4	****	51 ± 2.7	<	91 ± 3.7	****
EdU	99 ± 1.3	>	79 ± 6.1	****	99 ± 2.0	>	1.5 ± 1.0	****
PH3	5.1 ± 2.9	<	28 ± 6.3	**	8.9 ± 4.8	<	55 ± 22	****
PH3 / EdU	5.1 ± 3.0	<	19 ± 6.3	**	8.9 ± 4.8	>	0.4 ± 0.4	**
PH3 / EdU-neg	0.0 ± 0.0	<	9.6 ± 0.5	***	0.0 ± 0.0	<	55 ± 22	****

Fraction of cells (%; mean±SE) expressing indicated markers following treatment at the indicated time points (48 or 96 hours). Data based on three independent experiments. Statistical comparisons between the mock- or doxo-treated group: NS = p>0.05

** = p<0.01

*** = p<0.001

**** = p< 0.0001.

**Table 6:** Data set for IMR-32 *in vitro*.

	IMR-32 48h				IMR-32 96h			
	mock		doxo		mock		doxo	
Marker			1 μM	*p*			1+1 μM	*p*
TUNEL	21 ± 8.1	<	68 ± 5.2	****	17 ± 11	<	88 ± 2.2	****
c-casp3	2.3 ± 1.7	≃	1.6 ± 0.9	NS	6.2 ± 1.5	>	0.4 ± 0.3	***
Ki67	20 ± 13	≃	8.3 ± 10	NS	26 ± 19	>	4.6 ± 3.6	*
EdU	69 ± 24	>	18 ± 4.5	****	81 ± 18	>	12 ± 3.1	****
PH3	1.5 ± 0.4	≃	2.4 ± 1.4	NS	0.8 ± 0.4	<	3.0 ± 1.5	*
PH3 / EdU	0.8 ± 0.8	≃	0.5 ± 0.5	NS	0.4 ± 0.3	≃	0.3 ± 0.3	NS
PH3 / EdU-neg	0.7 ± 0.9	≃	1.8 ± 1.8	NS	0.4 ± 0.5	<	2.7 ± 1.3	*

Fraction of cells (%; mean±SE) expressing indicated markers following treatment at the indicated time points (48 or 96 hours). Data based on three independent experiments. Statistical comparisons between the mock- or doxo-treated group: NS = p>0.05

* = p<0.05

*** = p<0.001

**** = p< 0.0001.

#### Doxo-induced cell death

The doxo-resistant phenotype of BE(2)-C and doxo-sensitivity of IMR-32 was confirmed by TUNEL assay (Tables 5 and 6). Frequencies of early apoptosis (c-casp3) were not significantly affected by single or repeated exposure to doxo in BE(2)-C cells (Table 5). For IMR-32 cells, a single dose did not change the low frequency of c-casp3 positive cells, but a repeated exposure yielded a significant reduction of this marker (Table 6).

#### Effects on Ki67-index *in vitro*

Exposure of BE(2)-C cells to 1μM doxo resulted 48 hours later in a significant increase in the percentage of Ki67 positive cells (97±1.4 compared to mock-treatment 36±5.5; p<0.0001). Similar results were obtained after repeated doses, 1+1μM doxo, given with a 48-hour interval (p<0.0001,Table 5).

In IMR-32 cells, levels of Ki67 positive cells following 1μM doxo was not statistically different significant compared to mock-treatment (Table 6). However a significant reduction was observed following a repeated dosing (1+1μM doxo given with a 48-hour interval) (p<0.05,Table 6).

#### Induction of cell cycle arrest

To further analyse the cell cycle profiles of BE(2)-C and IMR-32 following doxo exposure we analysed the incorporation of 5-ethynyl-2-deoxyuridine (EdU), and/or fractions of cells staining positive for PH3 (Tables 5 and 6).

For BE(2)-C cells (Table 5); a single dose of 1μM doxo resulted in a decreased proportion of EdU positive cells (79±6.1 compared to mock-treatment 99±1.3, p<0.0001), as well as an increased proportion of PH3 positive cells (28±6.3% compared to mock-treatment 5.1±2.9%; p<0.01). The fraction of double positive (PH3+/EdU+) cells increased to 19±6.3%, compared to mock-treatment 5.1±3.0% (p<0.01). The remaining PH3 positive cells were EdU negative, i.e. growth arrested.

Repeating the dose (ie. 1+1μM doxo) with a 48-hour interval resulted in an almost complete ablation of EdU positive cells compared to mock-treatment (p<0.0001). The majority of PH3 positive cells were now EdU negative and only a minute fraction of PH3/EdU double positive cells (0.4±0.4%) was found, a significantly lower percentage compared to mock-treatment (8.9±4.8%; p<0.01).

For IMR-32 cells (Table 6); a single dose of 1μM doxo resulted in decreased levels of EdU positive cells (18±4.5% compared to mock-treatment 69±24%, p<0.0001). There was no difference in the percentages of PH3 positive cells in mock versus treated, which were both low. Similar to BE(2)-C cells repeated dose of 1+1μM doxo resulted in a decreased fraction of EdU positive cells (12±3.1% compared to mock-treatment 81±18%, p<0.001) for IMR-32. There was no difference in the percentages of PH3 positive cells in the mock-treated versus doxo-treated groups, where both values were low.

## Discussion

Chemotherapy (CT) is crucial for survival in high-risk NB and doxo is one of the most important drugs in highly active treatment in this patient group. In the present study, a dose dependent cytotoxic effect following doxo treatment was observed in the human embryonic PSCT-model, reiterating known characteristics of sensitive and multi-resistant phenotypes of two metastatic high-risk NB. Repeated doxo treatment was consistently more effective compared to an equivalent dose administered only once. This observation is in line with longstanding clinical experience of enhanced anti-tumour effects from repeated chemotherapy cycles [33].

Tumour-selective effects were indicated from the applied doxo regimens. Assessments of non-malignant highly proliferative tissues (developing embryonic neural epithelium, cartilage and muscle, located in the proximity of the studied NB-growth) revealed no increase in cytotoxic effects after doxo treatment compared to mock treatment. This finding is important for the analysis of anti-tumour effects in that it decreases, but not eliminates, the risk of confounding doxo-induced toxicity in the local microenvironment.

A somewhat unexpected finding in the PSCT model was that post doxo treatment the BE(2)-C tumour growth presented an increased Ki67-index (from 43% to 64%; p<0.01). Ki67 is a protein absent from non-cycling cells, strictly associated with cell proliferation and detectable in all active phases of the cell cycle [34], and thus the observation indicated a shift towards cycling cells induced by the exposure to doxo. This was dependent on repeated doxo treatment, possibly reflecting gradual tissue absorption of the drug [35]. Support for the notion that quiescent (G0) tumour cells entering cell cycle after doxo treatment was obtained also *in vitro*. The Ki67-index was investigated following exposure of cultured BE(2)-C cells to a pharmacologically relevant dose of doxo (1μM), allowing a uniform bioavailability of doxo in the culture medium. This resulted in a massive increase of Ki67 positive cells (97%; p<0.0001), >3-fold increase of PH3/EdU double positive cells (19%; p<0.001), however no cell death (0.2% TUNEL). Notably, also a reduction of EdU positive cells was observed (from 99% to 79%; p<0.0001) along with the appearance of EdU negative cells in G2/M-phase (9.6%; p<0.001), together indicating induction of cellular arrest, partly in G2/M-phase.

These findings are partly in line with previous reports that MYCN-amplified NB avoids arrest in G1- and/or S-phase, favouring a G2/M-phase enrichment and reduced cell death [36–41]. The results are consistent with findings in human hepatocellular carcinoma in which doxo was shown to accelerate cell cycle transition, at first allowing cell cycle continuation, but ultimately leading to cell cycle arrest [42]. Here it is also of potential interest that cells lacking a functional p53/p21 pathway have been shown to arrest in G2/M-phase through down regulation of cdk1 kinase activity by p14ARF [43].

Further investigations may elucidate whether the doxo-induced G2/M-arrested state in BE(2)-C is permanent or reversible. There might be a putative gain for the multi-resistant BE(2)-C cells to accumulate in G2/M-phase arrest. It has been shown that stable tetraploid clones are more resistant to chemotherapy-induced apoptosis than diploid counterparts, due to increased DNA repair and anti-apoptotic factors [44, 45].

A small number of studies have suggested enhanced tumour re-growth following chemotherapy [46]. Tumour growth/re-growth has been suggested to be stimulated through apoptosis‐induced proliferation, and diverse model systems have shown that apoptotic cells can secrete mitogens to directly stimulate cell proliferation. For example, *in vitro* studies have shown IGFII-like factor secretion from BE(2)-C cells into the culture medium, resulting in an autocrine/paracrine stimulation of DNA replication and cell growth [47]. In another type of study, long-term drug selection of BE(2)-C MYCN amplified NB cells with doxorubicin was shown to enrich for a cancer-stem-cell-like subpopulation [48]. Calcagno and colleagues reported a similar conclusion from *in vitro* studies of breast cancer [49]. Kurtova and colleagues showed that chemotherapy induces prostaglandin PGE_2_ in neighbouring cells, triggering cell division of putative cancer stem cell populations in bladder cancer [50].

However, a proliferative boost was not observed of IMR-32 tumours after doxo-therapy, neither *in vivo* nor *in vitro*. Exposure *in vitro* to 1μM doxo revealed instead extensive cell death (68% TUNEL positive). The combined *in vivo/in vitro* analysis thus indicated presence of a functionally active p53 pathway. Evidently, numerous factors, genetic and epigenetic, may relate to this difference but IMR-32 cells have been previously shown to express low levels of the p53 downstream target p21 and cells with low levels of p21 are more likely to enter apoptosis [37].

A repeated doxo exposure *in vitro* (1+1μM) further enhanced the differences between BE(2)-C and IMR-32 with regard to survival, possibly reflecting their difference in p53 status. The vast majority of BE(2)-C cells survived a second exposure (TUNEL 0.6%) and maintained positive staining for Ki67 (91%). However, most cells went into growth arrest (98% EdU negative), mainly in late G2/M-phase (55% PH3 positive). IMR-32 on the other hand exhibited high levels of growth arrest (88% EdU negative) but here leading to late apoptosis/cell death (TUNEL 88%). Only a small fraction of IMR-32 was arrested in late G2/M-phase following the repeated doxo-exposure (2.7% PH3 positive).

Doxorubicin-induced death has been reported to be independent of caspase in N-type NB cells (e.g. IMR-32) [51]. In our study a single doxo *in vivo* or *in vitro* exposure of IMR-32 did not alter the frequencies of c-casp3 positive cells. However, assessments of double exposures (4+4mg/kg i*n vivo* and 1+1μM *in vitro*) resulted in a significant increase of cleaved caspase-3 (p<0.01 in both cases). The reason for this discrepancy needs to be further explored.

## Conclusions

In summary, we have demonstrated the use of the human embryonic microenvironment in the PSCT model for *in vivo* evaluation of chemotherapy response in high-risk NB. The results are encouraging for the further development of clinically relevant studies of intra tumour heterogeneity and asynchronous tumour response to therapy in NB and other tumours originating early in life. Notably, a phenomenon of compensatory re-growth of resistant cells/clones following CT was detected for the multi-resistant p53 mutated NB tumour line BE(2)-C, but not for the drug-sensitive p53 wild type NB line IMR-32. Further investigations are needed to study the molecular regulation of arrest after recurrent treatment. Chemotherapy is long known as a double-edged sword and the new findings next need to be evaluated for generality and potential clinical relevance.
